# Archive Mining Brings to Light a 25-Year Old Astrovirus Encephalitis Case in a Sheep

**DOI:** 10.3389/fvets.2019.00051

**Published:** 2019-03-04

**Authors:** Leonore Küchler, Michel C. Koch, Torsten Seuberlich, Céline L. Boujon

**Affiliations:** ^1^Division of Experimental Clinical Research, Vetsuisse Faculty, University of Bern, Bern, Switzerland; ^2^Graduate School for Cellular and Biomedical Sciences, University of Bern, Bern, Switzerland; ^3^Institute of Social and Preventive Medicine, Lausanne University Hospital, Lausanne, Switzerland

**Keywords:** astrovirus, encephalitis, sheep, formalin-fixed and paraffin embedded (FFPE), next-generation sequencing (NGS)

## Abstract

In mammals, the small, positive-sense single-stranded RNA astroviruses are known as being mostly enterotropic and host-specific. Over the past years, however, they were identified several times in central nervous system tissues of humans, minks, cattle, sheep, and pigs with nonsuppurative inflammatory disease of that organ system. We recently reported such neurotropic astroviruses, amongst which bovine astrovirus CH15 (BoAstV-CH15) in two cows, and ovine astrovirus CH16 (OvAstV-CH16) in a sheep, which were genetically almost identical to one another. In order to investigate the occurrence of this virus species in Switzerland over time, we selected formalin-fixed, paraffin-embedded (FFPE) brain tissues of small ruminants diagnosed with severe encephalitis between 1969 and 2012 and screened those by immunohistochemistry for the capsid protein of BoAstV-CH15/OvAstV-CH16. We found one sheep, which died in 1992, that displayed positive immunostaining in various brain regions, and observed that immunostained cells were generally co-localized with the strongest histopathological lesions. We confirmed the virus presence with a second immunohistochemical protocol and demonstrated its close genetic relationship to other BoAstV-CH15/ OvAstV-CH16 strains by next-generation sequencing of an RNA extract from FFPE brain material. Our findings demonstrate that astrovirus BoAstV-CH15/OvAstV-CH16 existed in Switzerland already more than 2 decades ago and underline again the close relationship of the bovine and ovine strains of this virus.

## Introduction

*Astroviridae*, which are positive-sense, single-stranded RNA viruses, were described in a huge variety of birds and mammals (1). The genus *Avastrovirus* is known to infect birds, while mamastroviruses are to be found in mammalian hosts, in which they were identified mostly in fecal samples. The ca. 7 kb long genome of astroviruses displays three open reading frames (ORF): ORF1a and ORF1b (which is translated through a ribosomal frameshift mechanism together with ORF1a) encode nonstructural proteins, whereas capsid proteins are derived from ORF2 (1). Currently, the taxonomy of astroviruses is based on the host species from which they were isolated, as well as the amino acid sequence of the capsid protein precursor (2). Within the *Mamastrovirus* genus, astroviruses are thus classified within the same genotype species when the amino acid genetic distance of their capsid protein precursor sequence is <0.338. Astroviruses were long considered to be host-specific; however, several phylogenetic analyses questioned this assumption (3–5).

After astroviruses were observed by electron microscopy in fecal samples of children with gastroenteritis for the first time in 1975 (6, 7), an ovine equivalent of these viruses was soon identified in the feces of diarrheic lambs (8). Further molecular studies spoke for the existence of two enterotropic astrovirus genotype species in sheep (ovine astrovirus 1 and 2), which are genetically quite distant from each other (9, 10). From 2010 onwards, astroviruses were reported in association with different inflammatory diseases of the central nervous system in humans, minks, cattle and pigs (11–15). Ovine neurotropic astroviruses were first described in one ewe and one lamb belonging to the same herd in Wales, and that both died 9 months apart of nonsuppurative polioencephalomyelitis and dorsal root ganglionitis (16). We recently also reported one case of astrovirus encephalitis in a sheep, which we identified by next-generation sequencing (NGS) (17). Similarly to its Welsh counterparts, ovine astrovirus CH16 (OvAstV-CH16) was almost identical (>98 and >93% identity on the amino acid level, respectively) to bovine astrovirus CH15 (BoAstV-CH15) and bovine astrovirus BH89/14 (BoAstV-BH89/14), both of which were discovered in cattle with nonsuppurative inflammatory nervous disease in Switzerland and Germany, respectively (18, 19). Based on above-mentioned criteria, all these ovine and bovine neurotropic astroviruses would therefore belong to the same genotype species. In goats, on the other hand, there is no report of astroviruses to date, neither from stool samples nor from neurologically diseased animals. However, as BoAstV-CH15/OvAstV-CH16 seems to affect sheep just as well as cattle, it could be assumed that goats might also belong to the host spectrum.

The aim of this study was to investigate the occurrence of BoAstV-CH15/OvAstV-CH16 in Swiss small ruminants diagnosed with nonsuppurative meningoencephalitis over a period of more than 40 years. Using antibodies originally raised against BoAstV-CH15, we screened ovine and caprine brain tissue samples from our archive by immunohistochemistry (IHC). Subsequently, we investigated further one positive sheep with an additional antibody by IHC, as well as by qRT-PCR and NGS, in order to gain some insights into the genetics of the newly discovered virus.

## Materials and Methods

### Tissue Samples

Formalin-fixed, paraffin-embedded (FFPE) brain samples of small ruminants were available from the archive of the Division of Experimental Clinical Research, Vetsuisse Faculty, University of Bern (Bern, Switzerland). Forty-eight animals (34 sheep, 14 goats) with moderate to severe nonsuppurative meningoencephalitis indicative of viral infection but of unknown etiology, whose tissues were collected between 1969 and 2012, were selected. All animals were submitted to diagnostic neuropathological investigation after dying of sickness or being euthanized because of it, and approval for this study was therefore not required as per the local legislation. As some of the samples were stored for decades, certain tissues first had to be re-embedded in order to facilitate sectioning. Tissue sections of all brain regions available were stained with hematoxylin and eosin (H&E).

### IHC

Initially, two brain regions per animal were screened by IHC, with samples including the hippocampus, obex, or cerebellum being preferentially used. Subsequently, all brain regions available for the positive case were investigated. The immunohistochemical staining procedure was essentially the same as the one we described previously (17). Two kinds of polyclonal antibodies were obtained by immunizing rabbits with recombinant viral proteins (that were designed on the capsid protein sequence of BoAstV-CH15). IHC was performed as follows: tissue sections were deparaffinized, rehydrated, and endogenous peroxidase activity was blocked in a solution of 3% H_2_O_2_ in methanol. Subsequently, they were microwave cooked in Dako Target Retrieval Solution, Citrate pH 6 (Agilent) and blocked with 10% normal goat serum in PBS-T. The samples were incubated with the primary antibody CH15-ORF2-var (diluted 1:50 in PBS-T) and detection was carried out with Dako REAL Detection System, following the manufacturer's instructions. For the positive case, all brain regions were also tested with the primary antibody CH15-ORF2-con (17), using the same procedure as above. All samples were run in parallel with OvAstV-CH16-positive and -negative controls.

### RNA Extraction

RNA was extracted essentially as described in a study by Delnatte et al. (20). In brief, two 20 μm-thick sections of FFPE midbrain of sheep 21979 were deparaffinized with xylol and further processed with the RNeasy FFPE kit (Qiagen) according to the manufacturer's instructions. The positive control used in this study was extracted with TRI Reagent (Merck) from frozen brain tissue of our OvAstV-CH16 index case (ID 41669) (17).

### qRT-PCR

Three or 1 μl RNA extract from FFPE midbrain tissue of sheep 21979 or frozen brain tissue of sheep 41669, respectively, were investigated for the presence of BoAstV-CH15/OvAstV-CH16 sequences with the AgPath-ID RT-PCR kit (Thermo Fisher Scientific) according to the manufacturer's instructions, using the primers combination “CH15.” The latter was designed with Geneious 10.1.3 (https://www.geneious.com) based on the alignment of all BoAstV-CH15/OvAstV-CH16 sequences available (Bovine astrovirus isolate CH15: accession no. KT956903.2; Bovine astrovirus BH89/14: accession no. LN879482.1; Mamastrovirus 13 isolate UK/2013/ewe/lib01454: accession no. LT706531; Mamastrovirus 13 isolate UK/2014/lamb/lib01455: LT706530.1; Mamastrovirus 13 strain CH16: accession no. KY859988.1): 5′-CGTAGCACCCCCTTACAGCA-3′ (forward primer), 5′-CCTCGATCCTACTCGGCGTG-3′ (reverse primer), 5′-FAM-CTTAGAGGCCACGCAGAAGC-BHQ1-3′ (probe). The assay was run on a 7,300 Real-Time PCR system (Applied Biosystems) with following conditions: 45°C for 10 min, 95°C for 10 min, 40 cycles of 95°C for 15 s, 62°C for 20 s, 60°C for 30 s.

### NGS

Starting material for library preparation was 50 ng RNA extract from FFPE midbrain tissue of sheep 21979. A cDNA library was prepared with the SMARTer Stranded Total RNA-Seq Kit v2—Pico Input Mammalian (Takara Bio Inc.), without fragmentation and with repeated purifications with AMPure XP beads (Beckman Coulter). Before single-end sequencing (100 bp) on half a lane with a HiSeq 3000 System (Illumina), the library quality was controlled on a Qubit Fluorometer (ThermoFisher) and a Fragment Analyzer (Advanced Analytical Technologies Inc.) with the High Sensitivity NGS Fragment Analysis Kit (Advanced Analytical Technologies Inc.).

### *De novo* Assembly and Mapping

After quality trimming and subtraction of adapter sequences with fastp (version 0.12.5, parameters: –l 55 –W 4 –M 15 −5 3 −3 3) and removal of reads mapping to the ovine reference genome (Oar_v4.0) with STAR (version 2.6.0c, default parameters), remaining reads were assembled using SPAdes (version 3.12.0, parameters: –k 21,33,55). The resulting contiguous sequences (contigs) were aligned to entries of the RefSeq Viral Genome database completed with neurotropic astrovirus sequences (Bovine astrovirus isolate CH15; Bovine astrovirus BH89/14; Mamastrovirus 13 isolate UK/2013/ewe/lib01454; Mamastrovirus 13 isolate UK/2014/lamb/lib01455; Mamastrovirus 13 strain CH16) using BLASTN (Ver. 2.7.1+, default parameters). Contigs ≥ 100 nt long and aligning to astrovirus sequences were finally reassembled with Geneious 10.1.3 (DeNovo Assembly, parameters: “Low Sensitivity/Fastest”; https://www.geneious.com).

### Phylogenetic Analysis

The amino acid sequences of the capsid protein precursor of the new astrovirus strain and selected members of the family *Astroviridae* were used to construct a Maximum-Likelihood tree in MEGA7 (21), using the Whelan and Goldman model (22) with 1,000 bootstrap replicates. The model was selected thanks to the “Find Best DNA/Protein Models (ML)” option in MEGA7, based on the evaluation of 56 amino acid substitution models.

## Results

### One Sheep Showed Positivity for BoAstV-CH15/OvAstV-CH16 by IHC

**Figure 1:**
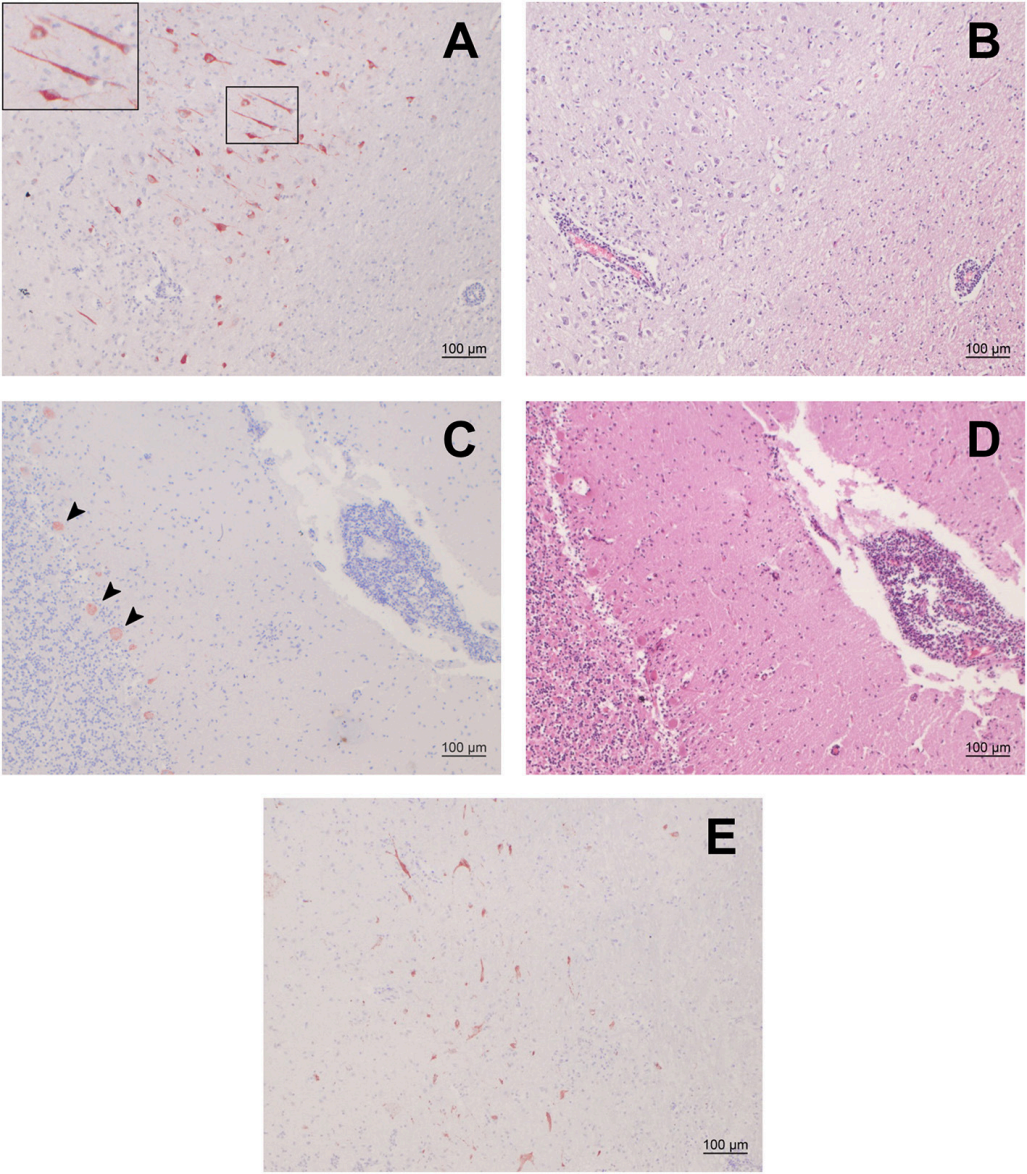
Immunohistochemistry (IHC) for BoAstV-CH15/ OvAstV-CH16 in sheep 21979 and corresponding histopathological lesions. IHC using hyperimmune antiserum CH15-ORF2-var **(A,C)** and hematoxylin-eosin staining of corresponding areas **(B,D)**. **(A)** Cerebral cortex; the inset on the upper left shows the marked area at a higher magnification. **(B)** Cerebral cortex; apparent perivascular cuff on the lower left. **(C)** Cerebellum; arrowheads point out positively-stained Purkinje cells. **(D)** Cerebellum; pronounced perivascular cuff on the right. **(E)** IHC using hyperimmune antiserum CH15-ORF2-con, showing positive staining in the diencephalon of sheep 21979.

We selected 48 small ruminants (34 sheep, 14 goats) with moderate to severe nonsuppurative encephalitis from our archive and screened FFPE brain samples of these animals by IHC for BoAstV-CH15/OvAstV-CH16 capsid antigen. The samples investigated originated from Swiss young and adult animals that were submitted to our neuropathological diagnostic service between 1969 and 2012. One sheep (ID 21979) showed positivity in various brain regions (Figure 1), whereas all the other animals were negative. The positive animal was a ewe that first displayed nervous behavior shortly before lambing. It then showed fever and ataxia and also appeared to have a foreign body stuck in its esophagus. It was finally submitted in March 1992 to our diagnostics services with suspicion of listeriosis. No further information about the age, breed or cause of death of the animal was available. Histopathologically, the animal presented massive polioencephalitis with mononuclear vascular infiltrates, gliosis and neuronal necrosis, the most important lesions being seen in the hippocampus, the thalamus and the medulla oblongata. As assessed morphologically, cells stained by the IHC procedure appeared to be neurons exclusively (Figure 1A). Compared to the first sheep found positive for OvAstV-CH16 in our laboratory (17), immunostained cells were visible in a more extensive manner, but the brain regions affected were similar. A second IHC protocol detecting another part of the capsid protein of BoAstV-CH15/OvAstV-CH16 was performed on all brain regions of animal 21979 in order to confirm the virus presence, and was also positive (Figure 1E).

### Immunopositive Cells Were Generally Co-localized With Histopathological Lesions

We compared the localization of immunostained cells with that of histopathological lesions in the brain regions available. Positive cells could be seen in the gray matter of all of them, with a variable density. In the occipital and parietal cortex, hippocampus, midbrain, brainstem, and rostral midbrain, immunopositive cells were observed where pronounced histopathological lesions were located. In the thalamus, on the other hand, surprisingly few cells showed positivity in regard to the severity of the lesions that were to be seen. In either case, however, immunopositive cells were always found in the vicinity of perivascular cuffs or gliosis foci (Figure 1).

### Genetic Data Demonstrates High Similarity of the Newly Discovered Virus With Other Ovine Neurotropic Astroviruses

**Figure 2:**
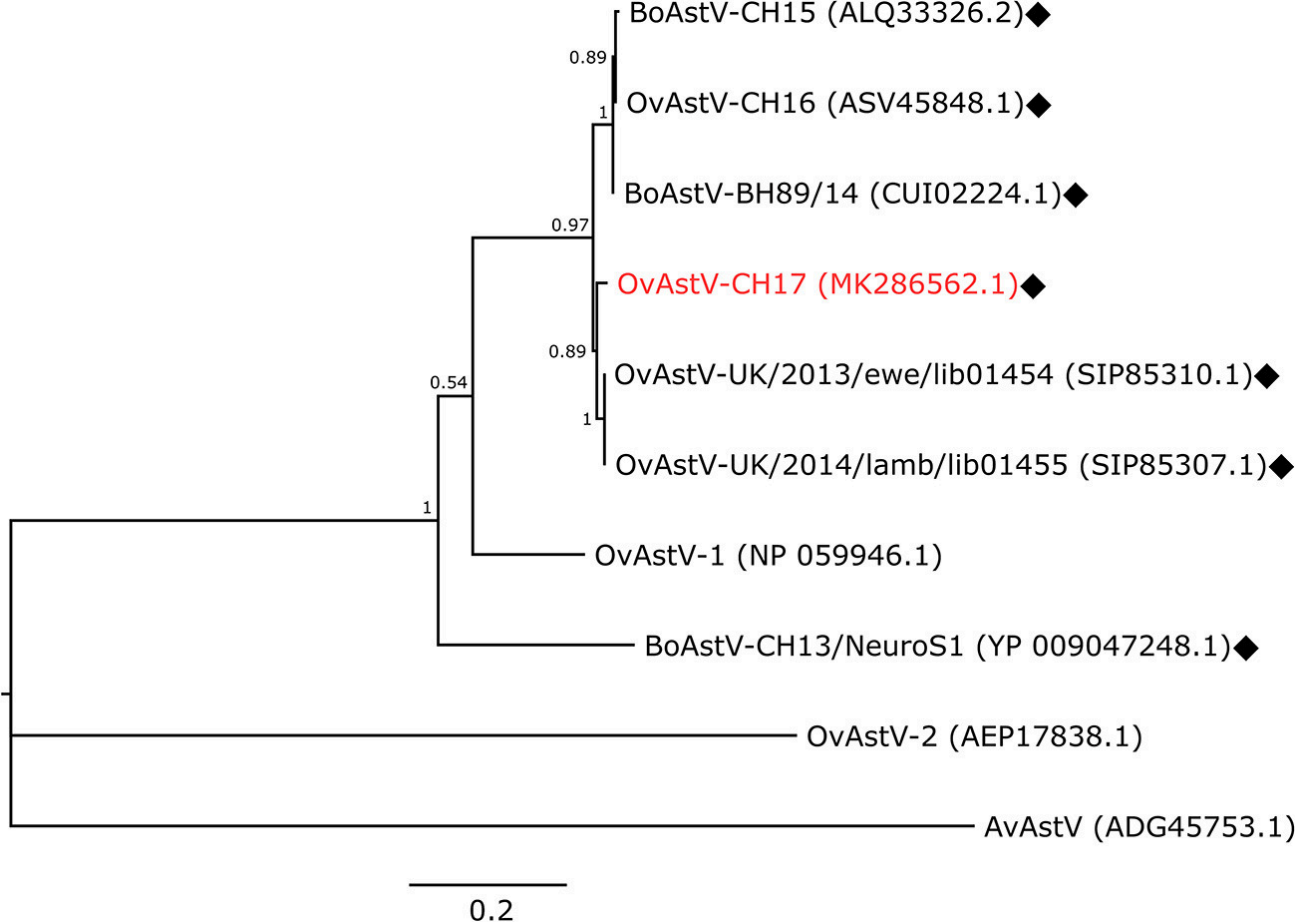
Phylogenetic analysis. Maximum-Likelihood tree constructed with the capsid protein precursor sequences of the new astrovirus strain OvAstV-CH17 and selected astroviruses. GenBank accession numbers are shown in brackets. Neurotropic strains are indicated with rhombi. AvAstV, avian astrovirus; BoAstV, bovine astrovirus; OvAstV, ovine astrovirus.

An RNA extract from FFPE midbrain tissue of animal 21979 was tested by qRT-PCR with primers specific for BoAstV-CH15/OvAstV-CH16. It scored positive with a cycle threshold (ct) value of 31, similar to that of an RNA extract from frozen brain material of our OvAstV-CH16 index case (ID 41669, ct = 29). We then submitted the RNA extract of animal 21979 to NGS, which resulted in the generation of 202,725,928 reads (European Nucleotide Archive accession number ERS3014183). After quality-trimming and removal of reads mapping to the host genome, we obtained 87 contigs that had a BLAST hit on an astrovirus sequence, on nucleotide and/or on amino acid level. Together, they constituted a 6,475 nt-long sequence (GenBank accession number MK286562.1) after reassembly. The characteristic features of the family *Astroviridae* were present, such as three open-reading frames (ORF), with a ribosomal frameshifting signal between the putative ORF1a and ORF1b. In comparison to most of the other strains of the BoAstV-CH15/OvAstV-CH16 cluster, there were three additional nucleotides at the 5′ end of the genome; however, as a RACE was not performed, this difference could not be confirmed. On the 3′ end of the genome, a series of adenines corresponded to the virus's poly(A) tail. The new astrovirus strain demonstrated high identity to members of the BoAstV-CH15/OvAstV-CH16 group: 98.7% to BoAstV-CH15 for the putative translation product of ORF1ab, 97.8 % to OvAstV-UK/2014/lamb/lib01455 (16) for the capsid protein precursor (Table 1). A phylogenetic analysis based on the capsid protein precursor sequence of different astroviruses (Figure 2) showed that the closest relatives of the virus were ovine neurotropic astroviruses from Scotland (16) and confirmed its belonging to the BoAstV-CH15/OvAstV-CH16 cluster.

**Table 1:** Pairwise sequence similarities [%] of selected astrovirus strains in ruminants.

	**OvAstV-1**	**BoAstV-CH13**	**BoAstV-CH15**	**BoAstV-BH89/14**	**OvAstV-UK/ 2013/ewe**	**OvAstV-UK/ 2014/lamb**	**OvAstV-CH16**	**OvAstV-CH17**
**ORF1ab / nsp1ab**								
OvAstV-1	–	61.36	76.42	76.57	76.49	76.57	76.64	76.64
BoAstV-CH13	61.75	–	60.54	60.61	60.47	60.47	60.54	60.54
BoAstV-CH15	70.50	63.29	–	99.04	98.67	98.60	99.41	98.67
BoAstV-BH89/14	70.40	63.43	91.98	–	98.89	98.97	98.89	98.53
OvAstV-UK/2013/ewe	70.35	63.70	91.80	94.11	–	99.93	98.38	98.30
OvAstV-UK/2014/lamb	70.38	63.72	91.78	94.13	99.98	–	98.45	98.38
OvAstV-CH16	70.56	63.19	98.63	92.10	92.04	92.07	–	98.60
OvAstV-CH17	70.33	62.73	91.29	90.70	91.23	91.26	91.60	–
***ORF2 / capsid***								
OvAstV-1	–	66.28	72.01	72.53	73.05	72.92	72.27	73.18
BoAstV-CH13	64.24	–	64.07	64.33	63.94	63.94	64.46	63.68
BoAstV-CH15	70.03	65.37	–	98.95	94.99	94.86	99.34	94.47
BoAstV-BH89/14	70.11	65.63	94.91	-	95.13	94.99	99.34	94.73
OvAstV-UK/2013/ewe	70.37	64.60	88.38	87.86	-	99.87	95.13	97.76
OvAstV-UK/2014/lamb	70.28	64.60	88.29	87.86	99.91	–	94.99	97.63
OvAstV-CH16	70.03	65.63	99.30	94.74	88.25	88.16	–	94.60
OvAstV-CH17	69.55	65.20	86.45	86.63	88.42	88.42	86.41	–

*ORF, open reading frame; nsp1ab, nonstructural protein 1ab; capsid, capsid protein precursor. White cells, nucleotide similarity; gray cells, amino acid similarity. OvAstV-UK/2013/ewe, OvAstV-UK/2013/ewe/lib01454; OvAstV-UK/2014/lamb, OvAstV- UK/2014/lamb/lib01455*.

## Discussion

Recently, astroviruses were reported in three sheep with different nonsuppurative inflammatory patterns of the central nervous system: one ewe and one lamb belonging to the same herd in Wales (16), as well as a single case from Switzerland (17). All these viruses were genetically very close to one another and clustered tightly together with two other bovine neurotropic astroviruses (18, 19). We report here a further case of astrovirus encephalitis in a sheep (ID 21979), which we discovered by screening archived brain tissues of 48 small ruminants by IHC for the capsid protein of the astrovirus genotype species involved in the cases mentioned above. NGS of an RNA extract from FFPE tissue of the positive animal confirmed a very high identity of the nucleic acid sequence of the strain described here, tentatively named ovine astrovirus CH17 (OvAstV-CH17), to the other neurotropic ovine and bovine astroviruses cited previously, showing its close relationship to these viruses, not only antigenically but also genetically.

First, these findings demonstrate that our recently developed IHC procedure is an appropriate tool for screening material of neurologically-diseased animals, even after long-term storage. On the other hand, it is very specific and does not exclude that divergent astrovirus genotype species could be implicated in other cases of nonsuppurative inflammatory disease of the central nervous system in small ruminants. Indeed, in humans, cattle, and pigs, various astrovirus genotype species have been found to be associated with such illnesses (13–15, 19, 23). Still, as our antibodies were originally raised against BoAstV-CH15 (that we identified in two cases of encephalitis in cattle), their reactivity in the sheep of this study as well as in another previously reported case (17) underlines again a close genetic relationship between these specific ovine and bovine astroviruses, further increasing the plausibility of a transmission of the virus between both host species.

Because of cross-linking and fragmentation due to exposition to formalin, RNA extracts from FFPE tissues are often assumed to be difficult targets for nucleic acids-based assays (24). Yet, we were able to detect viral RNA in sheep 21979 by qRT-PCR and obtain almost the full genome length of the virus by NGS. These successful outcomes might be due to the short length of the target amplicon of our qRT-PCR (116 bp) and of reads (100bp) chosen for NGS.

As the astrovirus-positive sheep in this study died in 1992, our findings also demonstrate that virus BoAstV-CH15/OvAstV-CH16 was present in Switzerland already more than two decades ago; the other bovine and ovine cases dated back to 2002, 2006–2007, and 2013–2014, a fact that tends to show that the virus was circulating in Europe during all this time, although at a very low rate. This last aspect contrasts with the data about bovine astrovirus CH13/NeuroS1 (BoAstV-CH13/NeuroS1), another genotype species that could be found in a high percentage (up to 85%) of cattle with nonsuppurative patterns of neurological diseases (13, 25–29). Still, as nonsuppurative inflammatory patterns in the nervous system are often indicative of a viral infection, the etiology of the majority of the cases in our study is still pending and requires further research. As we were able to detect astroviral RNA from FFPE tissue by qRT-PCR in sheep 21979, such an approach would represent a further possibility to investigate these etiologically unresolved cases, also for other pathogens. Moreover, since NGS of an RNA extract of sheep 21979 was successful, this method could be used to this end as well.

A detailed investigation of the repartition of immunoreactive cells in sheep 21979 tended to show a high co-localization with the most severe histopathological lesions in the different nervous tissues available. On the other hand, the absence of stained cells in some areas with pronounced lesions could be explained by a potential clearance of the virus, and therefore immunohistochemical target, in the immune reaction process. Yet, together with similar findings in the other astrovirus-positive sheep that we identified (17), this supports an association and even a causal link of the virus with the disease; however, a larger number of cases would be necessary to strengthen this assertion. In cattle, an extensive study about the co-localization of the distinct neurotropic astrovirus genotype species BoAstV-CH13/NeuroS1 with histopathological lesions (28) also showed a good correlation in most of the cases investigated. Lastly, propagation in cell culture or *in vivo* inoculation studies are still missing in order to demonstrate infectivity and pathogenicity of both these astrovirus genotype species. Further studies about small ruminants astroviruses, including those associated with enteric infections, might also shed light upon this.

Unfortunately, no tissues apart from those of the central nervous system of sheep 21979 were available for further investigation, thus preventing insights into the disease pathogenesis. Apart from immunosuppression, which seems to play an important role in humans, the factors contributing to neuroinvasion by astroviruses in other species (minks, cattle, sheep, and pigs) remain unknown. Nonetheless, as the host specificity of BoAstV-CH15/OvAstV-CH16 appears to be ambiguous and several other studies speak for interspecies transmission events of astroviruses, also involving humans (3, 4, 30, 31), zoonotic potential cannot be excluded, also in the context of neurological diseases.

## Data Availability

The dataset generated and analyzed in this study can be found in the European Nucleotide Archive (Accession No. ERS3014183). The assembled viral sequence is available in GenBank under accession number MK286562.1.

## Author Contributions

TS and CB conceived the experiments. LK and CB carried out the experiments. MK performed the bioinformatics analysis. LK, CB, MK, and TS analyzed the data. CB and MK wrote the article, TS edited it.

### Conflict of Interest Statement

The authors declare that the research was conducted in the absence of any commercial or financial relationships that could be construed as a potential conflict of interest.

## Abbreviations

BoAstV-CH13/NeuroS1: bovine astrovirus CH13/NeuroS1
BoAstV-CH15: bovine astrovirus CH15
FFPE, formalin-fixed: paraffin-embedded
IHC: immunohistochemistry
NGS: next-generation sequencing
OvAstV-CH16: ovine astrovirus CH16
OvAstV-CH17: ovine astrovirus CH17.

## References

[1] Bosch A Pinto RM Guix S. Human astroviruses. Clin Microbiol Rev. (2014) 27:1048–74. 10.1128/CMR.00013-14 25278582 PMC4187635

[2] Bosch A Guix S Krishna NK Méndez E Monroe SS Pantin-Jackwood M Family—Astroviridae. In: King AMQ Lefkowitz E Adams MJ Carstens EB editors. Virus Taxonomy: Ninth Report of the International Committee on Taxonomy of Viruses. San Diego, CA: Elsevier (2012). p. 953–9.

[3] Chu DK Chin AW Smith GJ Chan KH Guan Y Peiris JS. Detection of novel astroviruses in urban brown rats and previously known astroviruses in humans. J Gen Virol. (2010) 91(Pt 10):2457–62. 10.1099/vir.0.022764-0 20554799 PMC3052596

[4] Rivera R Nollens HH Venn-Watson S Gulland FM Wellehan JF Jr. Characterization of phylogenetically diverse astroviruses of marine mammals. J Gen Virol. (2010) 91(Pt 1):166–73. 10.1099/vir.0.015222-0 19759240

[5] Nagai M Omatsu T Aoki H Otomaru K Uto T Koizumi M. Full genome analysis of bovine astrovirus from fecal samples of cattle in Japan: identification of possible interspecies transmission of bovine astrovirus. Arch Virol. (2015) 160:2491–501. 10.1007/s00705-015-2543-7 26212364

[6] Appleton H Higgins PG. Letter: viruses and gastroenteritis in infants. Lancet. (1975) 1:1297. 10.1016/S0140-6736(75)92581-7 48925

[7] Madeley CR Cosgrove BP. Letter: 28 nm particles in faeces in infantile gastroenteritis. Lancet. (1975) 2:451–2. 10.1016/S0140-6736(75)90858-2 51251

[8] Snodgrass DR Gray EW. Detection and transmission of 30 nm virus particles (astroviruses) in faeces of lambs with diarrhoea. Arch Virol. (1977) 55:287–91. 10.1007/BF01315050 413529

[9] Jonassen CM Jonassen TT Sveen TM Grinde B. Complete genomic sequences of astroviruses from sheep and turkey: comparison with related viruses. Virus Res. (2003) 91:195–201. 10.1016/S0168-1702(02)00269-1 12573498

[10] Reuter G Pankovics P Delwart E Boros A. Identification of a novel astrovirus in domestic sheep in Hungary. Arch Virol. (2012) 157:323–7. 10.1007/s00705-011-1151-4 22033597 PMC3518301

[11] Blomstrom AL Widen F Hammer AS Belak S Berg M. Detection of a novel astrovirus in brain tissue of mink suffering from shaking mink syndrome by use of viral metagenomics. J Clin Microbiol. (2010) 48:4392–6. 10.1128/JCM.01040-10 20926705 PMC3008476

[12] Quan PL Wagner TA Briese T Torgerson TR Hornig M Tashmukhamedova A. Astrovirus encephalitis in boy with X-linked agammaglobulinemia. Emerg Infect Dis. (2010) 16:918–25. 10.3201/eid1606.091536 20507741 PMC4102142

[13] Li L Diab S McGraw S Barr B Traslavina R Higgins R. Divergent astrovirus associated with neurologic disease in cattle. Emerg Infect Dis. (2013) 19:1385–92. 10.3201/eid1909.130682 23965613 PMC3810933

[14] Arruda B Arruda P Hensch M Chen Q Zheng Y Yang C. Porcine Astrovirus type 3 in central nervous system of swine with polioencephalomyelitis. Emerg Infect Dis. (2017) 23:2097–100. 10.3201/eid2312.170703 29148383 PMC5708247

[15] Boros A Albert M Pankovics P Biro H Pesavento PA Phan TG. Outbreaks of neuroinvasive astrovirus associated with encephalomyelitis, weakness, and paralysis among Weaned Pigs, Hungary. Emerg Infect Dis. (2017) 23:1982–93. 10.3201/eid2312.170804 29148391 PMC5708238

[16] Pfaff F Schlottau K Scholes S Courtenay A Hoffmann B Hoper D. A novel astrovirus associated with encephalitis and ganglionitis in domestic sheep. Transbound Emerg Dis. (2017) 64:677–82. 10.1111/tbed.12623 28224712

[17] Boujon CL Koch MC Wuthrich D Werder S Jakupovic D Bruggmann R. Indication of cross-species transmission of astrovirus associated with encephalitis in sheep and cattle. Emerg Infect Dis. (2017) 23:1604–8. 10.3201/eid2309.170168 28820378 PMC5572871

[18] Schlottau K Schulze C Bilk S Hanke D Hoper D Beer M. Detection of a novel bovine astrovirus in a cow with encephalitis. Transbound Emerg Dis. (2016) 63:253–9. 10.1111/tbed.12493 26948516

[19] Seuberlich T Wuthrich D Selimovic-Hamza S Drogemuller C Oevermann A Bruggmann R. Identification of a second encephalitis-associated astrovirus in cattle. Emerg Microbes Infect. (2016) 5:e5. 10.1038/emi.2016.5 26785943 PMC5603821

[20] Delnatte P Ojkic D Delay J Campbell D Crawshaw G Smith DA. Pathology and diagnosis of avian bornavirus infection in wild Canada geese (Branta canadensis), trumpeter swans (Cygnus buccinator) and mute swans (Cygnus olor) in Canada: a retrospective study. Avian Pathol. (2013) 42:114–28. 10.1080/03079457.2013.769669 23581438

[21] Kumar S Stecher G Tamura K. MEGA7: molecular evolutionary genetics analysis version 7.0 for bigger datasets. Mol Biol Evol. (2016) 33:1870–4. 10.1093/molbev/msw054 27004904 PMC8210823

[22] Whelan S Goldman N. A general empirical model of protein evolution derived from multiple protein families using a maximum-likelihood approach. Mol Biol Evol. (2001) 18:691–9. 10.1093/oxfordjournals.molbev.a003851 11319253

[23] Vu DL Bosch A Pinto RM Guix S. Epidemiology of classic and novel human astrovirus: gastroenteritis and beyond. Viruses. (2017) 9:33. 10.3390/v9020033 28218712 PMC5332952

[24] Srinivasan M Sedmak D Jewell S. Effect of fixatives and tissue processing on the content and integrity of nucleic acids. Am J Pathol. (2002) 161:1961–71. 10.1016/S0002-9440(10)64472-0 12466110 PMC1850907

[25] Bouzalas IG Wuthrich D Walland J Drogemuller C Zurbriggen A Vandevelde M. Neurotropic astrovirus in cattle with nonsuppurative encephalitis in Europe. J Clin Microbiol. (2014) 52:3318–24. 10.1128/JCM.01195-14 24989603 PMC4313157

[26] Selimovic-Hamza S Bouzalas IG Vandevelde M Oevermann A Seuberlich T. Detection of astrovirus in historical cases of european sporadic bovine encephalitis, Switzerland 1958–1976. Front Vet Sci. (2016) 3:91. 10.3389/fvets.2016.00091 27781208 PMC5058262

[27] Wuthrich D Boujon CL Truchet L Selimovic-Hamza S Oevermann A Bouzalas IG. Exploring the virome of cattle with non-suppurative encephalitis of unknown etiology by metagenomics. Virology. (2016) 493:22–30. 10.1016/j.virol.2016.03.009 26994586

[28] Selimovic-Hamza S Boujon CL Hilbe M Oevermann A Seuberlich T. Frequency and pathological phenotype of bovine astrovirus CH13/NeuroS1 infection in neurologically-diseased cattle: towards assessment of causality. Viruses. (2017a) 9:E12. 10.3390/v9010012 28106800 PMC5294981

[29] Selimovic-Hamza S Sanchez S Philibert H Clark EG Seuberlich T. Bovine astrovirus infection in feedlot cattle with neurological disease in western Canada. Can Vet J. (2017b) 58:601–3. 28588333 PMC5432150

[30] Ulloa JC Gutierrez MF. Genomic analysis of two ORF2 segments of new porcine astrovirus isolates and their close relationship with human astroviruses. Can J Microbiol. (2010) 56:569–77. 10.1139/W10-042 20651856

[31] Karlsson EA Small CT Freiden P Feeroz MM Matsen FAt. Non-human primates harbor diverse mammalian and avian astroviruses including those associated with human infections. PLoS Pathog. (2015) 11:e1005225. 10.1371/journal.ppat.1005225 26571270 PMC4646697

